# The body does not keep the score: trauma, predictive coding, and the restoration of metastability

**DOI:** 10.3389/fnsys.2026.1812957

**Published:** 2026-04-30

**Authors:** Steven Kotler, Michael Mannino, Glenn Fox, Karl Friston

**Affiliations:** 1 Florida Atlantic University, Boca Raton, FL, United States; 2 Miami Dade College, Miami, FL, United States; 3 University of Southern California, Los Angeles, CA, United States; 4 University College London, London, United Kingdom

**Keywords:** active inference, free energy principle, metastability, post-traumatic stress disorder (PTSD), predictive processing

For nearly a decade, the idea that “the body keeps the score” has shaped public and clinical understanding of trauma (van der Kolk, 2014). It is an enticing metaphor—implying that experience is literally inscribed in flesh, that the body bears the scars of what the mind cannot face. Yet recent advances in computational and systems neuroscience reveal that this image, while emotionally compelling, is biologically inaccurate. The body proper does not store trauma; the brain dynamically reenacts it through maladaptive inference. What endures after trauma is not a memory lodged in non-innervated tissue but a collapse of flexibility—a loss of *metastability*, the brain's ability to fluidly switch among semi-stable network states.

In computational terms, trauma over-weights the precision of danger priors: the brain assigns excessive confidence to threat predictions, constraining inference based on the prior premise of enduring danger. The result is hypervigilance, flashbacks, and avoidance—symptoms of a system caught in self-confirming predictions. Mathematically, this overconfidence means one cannot escape local minima—in a free energy landscape—that become deeply and precisely engrained (i.e., trapped in a ravine with steep sides, where precision corresponds to the local curvature or steepness).

This rigidity contrasts with metastable dynamics, in which neural networks transiently integrate and segregate as conditions change. Using empirical and computational approaches, Hellyer et al. (2015) found reduced metastability following traumatic brain injury, associated with structural connectome damage, diminished cognitive flexibility, and impaired information processing. These findings concern physical brain injury, not psychological trauma or PTSD. Yet they suggest a question: could persistent threat also narrow the brain's repertoire of available states? We propose this as a theoretical extension. Its relevance to PTSD depends on testing whether altered metastable dynamics accompany persistent symptoms and whether recovery involves a return of context-sensitive flexibility.

The landscape in question is a free energy landscape where every belief is equipped with a measure of its plausibility (Friston, 2010). In this view, making sense of the world, and our bodies, entails a process of (Bayesian) belief updating that can be read as minimizing free energy or, in the vernacular, finding explanations for our sensations that contain the least surprise and the most plausibility.

Functional imaging studies illuminate this shift. In post-traumatic stress disorder (PTSD), the dominant neuroimaging finding is a dissociation: amygdala hyperactivation coupled with medial prefrontal hypoactivation, with mPFC responsivity inversely associated with symptom severity (Shin et al., 2006). The literature reveals heightened connectivity within threat-detection circuits alongside weakened top-down regulatory connections. An MEG study, for instance, found hyperconnectivity during threatening-face processing in posterior cingulate and right parietal regions, with increased node strength and clustering in the right amygdala and medial prefrontal cortex, suggesting reorganization rather than blanket rigidity (Dunkley et al., 2016). The net effect, however, converges on a reduction in the brain's dynamic repertoire: connectivity patterns shift toward defensive configurations, and the system's capacity for flexible reconfiguration is diminished.

From this view, trauma is a disorder of *prediction*, not *storage*; and thus why we believe it is inaccurate to say the body is somehow “keeping score.” Predictive coding reframes perception as active inference: the brain does not passively register the world but actively predicts it, adjusting only when errors arise, or acting to resolve errors that could not be solved without predictive coding. For example, a reflexive movement fulfills the brains predictions that our limbs should be in a particular place; thereby minimizing prediction error (Spoelstra et al., 2000), and reducing free energy. However, the brain can also resolve prediction errors by affording them less precision. A process known as sensory attenuation. In trauma, or ability to attenuate sensory precision is lost and prediction errors are mis-weighted. Internal threat expectations dominate the search for—and attention to—sensory evidence of danger; unattenuated interoceptive signals (racing heart, tight chest, etc.) are interpreted as confirmation of danger rather than imprecise noise. The “score” the body appears to keep is thus an artifact of circular inference: the brain predicts pain, senses arousal, and takes that arousal as proof that pain persists. The body participates in trauma, but as messenger, not archive.

This dynamic interpretation aligns with the broader field of embodied cognition. The body and environment are extensions of the brain's predictive loop, scaffolding thought through action. Yet embodiment is active and transient—it does not imply storage. Trauma-related somatic symptoms are better understood as mis-calibrated feedback between prediction, action, and sensation, not as remnants of the past frozen in muscle or fascia. The distinction matters. Where the storage model leads to metaphors of exorcism: finding and purging what was buried. By contrast, the inference model leads to training: recalibrating precision, retraining expectations, and expanding the brain's capacity for adaptive variability.

A more compelling thesis—for how emotional maps are truly embodied—comes from Antonio Damasio's Somatic Marker Hypothesis (SMH). The central premise of the SMH is that while the body provides the territory for emotion, the “maps” (e.g. the storage and representation of emotional experience) are constructed in the nervous system through distributed processing centers, including visceral, brainstem, and cortical networks.

Mechanistically, the re-emergence of a strong feeling—central to the experience of PTSD—can be explained using Damasio's notion of convergence-divergence zones (CDZs) (Damasio, 1989; Meyer and Damasio, 2009). CDZs are neural hubs that integrate and store the parameters for a memory and its associated emotion. They do not encode the raw sensory activations themselves, but rather coordinate the reactivation of these patterns in a flexible, context-dependent manner. In PTSD, one could hypothesize that CDZs become overly sensitive or “primed” to a wide variety of cues, thus triggering the cascade of conscious experiences related to trauma, even when the cues are only distantly related (for example, a car backfiring triggering a traumatic flashback).

The Somatic Marker Hypothesis, combined with the concept of convergence-divergence zones (CDZs), offers a robust explanation of trauma and the body through its emphasis on neural plasticity. Emotions and their bodily correlates are modifiable and adaptive, without requiring any notion of hidden storage in non-innervated bodily tissue. A fair assessment of van der Kolk's work requires acknowledging its considerable scope: The Body Keeps the Score explicitly (and commendably) discusses prefrontal-limbic interactions, interoception, embodied cognition, and draws substantially on Damasio's somatic marker framework. However, somatic markers are ultimately part and parcel of the brain's sophisticated ability to re-create states in the body—always originating in neural processes.

What van der Kolk gets right is his emphasis on the role of somatic states in trauma, reminding us that trauma is not just “in the head.” The paradox of the work is that it unintentionally reinforces the very mind-body duality that its core theses aim to dissolve: the notion that there is any territory of the body not connected to the brain and subject to the mind's renderings, and vice versa.

Van der Kolk is not naïve about neural substrates. Our concern is not with his invocation of neuroscience, but with the rhetorical emphasis of the work and, more importantly, its reception. The book's most memorable language—that trauma “lives in the body”—has often been taken fully and literally, suggesting that traumatic experience is stored in bodily tissues apart from neural innervation. This is a reading that the metaphor invites, even if the text occasionally qualifies it.

As illustration, van der Kolk describes the process of memory as having both rational and emotional systems (The Body Keeps the Score, 2014, Chapter 11: “Uncovering Secrets: The Problem of Traumatic Memory”). During trauma, he states, the rational system “disconnects other brain areas necessary for the proper storage and integration of incoming information, such as the hippocampus and thalamus.” The implication is that traumatic experiences tips the scale toward an “emotional” memory system that both fractures memories, and buries them deep in bodily tissue, faroutside the mind's purview. Indeed, Damasio's most cited book, *Descartes Error*, outlines the ways in which we should not treat “rational” and “emotional” processes as separate, but rather fully integrated and complementary to one another (Damasio, 1994).

Memories can emerge after years of being non-remembered, but the experience of remembering and the accuracy of what is remembered are different questions. Geraerts et al. (2007) found that continuously remembered childhood sexual abuse and memories unexpectedly recalled outside therapy were more likely to be corroborated than anticipated memories recovered in therapy. These findings invite caution about the circumstances of recall. They do not demonstrate that suggestion produces somatic feelings of trauma, and lack of corroboration does not establish that a particular memory is false. For the present argument, the distinction matters: the intensity of a bodily response cannot, by itself, settle the historical accuracy of the experience it accompanies.

Van der Kolk's framing obscures the central role of the nervous system in constructing, maintaining, and—crucially—transforming emotional experience. It can inadvertently imply that somatic intervention is the primary or only path to healing, sidelining the computational and inferential processes through which the brain generates the body's apparent “score.” Our argument is therefore less an outright refutation of van der Kolk than a updated reframing: the circular causality that underwrites the action-perception cycle—and the virtuous and vicious cycles that ensue—demands a more precise account of where trauma resides and how it can be resolved (Allen et al., 2022).

Resilience research provides further evidence that comports more naturally with an inference model than a storage model. George Bonanno and others have shown across large longitudinal cohorts that the majority of trauma-exposed individuals do not develop chronic PTSD (Bonanno, 2004). Most adapt. A review and statistical evaluation of 54 studies found resilience to be the most common trajectory, averaging 65.7% across the populations examined (Galatzer-Levy et al., 2018). Bonanno's data reveal multiple trajectories—resilience, recovery, delayed distress—but the resilient path is most common. To be clear, these data do not logically disprove a somatic storage account; a storage model could, in principle, accommodate individual differences in vulnerability. However, the prevalence of spontaneous recovery is more parsimoniously explained by an active inference framework: under typical conditions, the brain rebalances its predictive architecture, restoring metastability and flexibility without therapeutic intervention. Pathology arises not from the permanence of what was stored but from the failure of that recalibration process.

The practical question becomes: how can flexibility be restored when it is lost? Evidence now points to a surprising candidate—flow states. Flow, the state of complete absorption in a meaningful, high-challenge activity, may produce the very network dynamics that PTSD disrupts. During flow, the prefrontal cortex undergoes localized transient hypofrontality—reduced self-referential processing—while frontoparietal and sensory-motor networks exhibit rapid, adaptive reconfiguration. Functional MRI and EEG studies have implicated attentional, executive, and reward systems in flow, though the literature remains methodologically heterogeneous, with some findings supporting the transient hypofrontality hypothesis and others reporting increased prefrontal activity during challenge-skill balance conditions (Alameda et al., 2022). On theoretical grounds, we hypothesize that flow increases, and restores global metastability—that the brain's networks become more variable and context-responsive—but direct empirical confirmation using established metastability metrics (e.g., the Kuramoto order parameter) has not yet been achieved. This theoretical bridge, while compelling, should be understood as a prediction of the framework rather than an established fact.

(side note: in TBKTS, van der Kolk cites a woman who had severe surgical trauma that was untreatable by CBT, but psychodynamic therapy combined with Pilates provided a safe refuge and platform for recovery. Perhaps a complementary interpretation of this anecdote could posit the role that flow states could have played in her commendable recovery).

Theoretical models predict that neuromodulators such as dopamine, norepinephrine, and anandamide surge during flow (Kotler et al., 2022), heightening motivation, learning, and synaptic plasticity—though direct neurochemical measurement during naturalistic flow remains limited. If these predictions hold, flow returns the brain to the edge of criticality—the same flexible regime that trauma destroys.

Activity-based interventions offer a setting in which to investigate this possibility. Walter et al. (2023) randomized 96 active duty service members with major depressive disorder to 6 weeks of surf or hike therapy. Both groups improved over time, and surf participants were more likely to achieve remission at 3-month follow-up, although the groups did not differ significantly at post-program. These findings are encouraging, but the clinical change does not identify its mechanism. The trial did not measure metastability or test flow as a mediator, and concurrent treatment was permitted. Exercise, nature exposure, and social connection remain possible contributors. Whether moments of deep absorption add something specific to that process is a question for future research; this trial does not precisely establish a flow-based mechanism of recovery in PTSD.

Additionally, this process may hinge on a fundamental shift from avoidance to approach dynamics (Kotler et al., 2022). In the moments following threat detection, the nervous system can either constrict around defensive inhibition—freezing, bracing, retreating—or expand toward goal-directed engagement (i.e., more cognitive control). Approach behavior recruits dopaminergic and prefrontal systems that convert arousal into focused action (that rests upon sensory attenuation), while avoidance amplifies amygdala-driven threat signaling and motor suppression (that could be pathological in failures of sensory attenuation). Flow arises when the system interprets uncertainty as a call to act rather than a cue to withdraw, transforming fear into agency. In this sense, trauma and flow represent divergent adaptations to the same physiological stress signal—one collapses movement, the other restores it.

Cognitive-control research offers another way to understand why unwanted thoughts persist. Bomyea and Amir (2011) randomly assigned undergraduate participants to working-memory training with high or low inhibitory-control demands. Those in the high-demand condition showed greater improvement in working-memory performance and fewer intrusions during a subsequent thought-suppression task. The finding suggests that strengthening inhibitory control can change the persistence of unwanted thoughts in this experimental setting; it does not establish the cause of intrusive memories in PTSD. In the language of our framework, it raises a possibility: stronger control might give the system greater freedom to revise entrenched threat expectations. Whether this process alters metastability in PTSD remains an empirical question.

As stated above, neurodynamic models of flow (Kotler et al., 2022) clarify this further, showing that both trauma and flow begin with the same salience-network activation but diverge depending on the integrity of cognitive control. In flow, metastable coordination across the dorsal anterior cingulate cortex, insula, and ventromedial prefrontal cortex enables *higher cognitive control*: a state of dynamic stability where perception, emotion, and action merge seamlessly under flexible top-down guidance. In PTSD, this metastability collapses—network synchrony fragments, prefrontal inhibition weakens, and subcortical threat circuits dominate. Crucially, research shows that when individuals with PTSD train cognitive control—through mindfulness, working memory practice, or executive-function exercises—symptoms decrease and regulation improves. The “body's score,” then, is not a record of trauma, but a reflection of disrupted control [of sensory precision]; and thus, healing lies in restoring metastable coherence and reestablishing the brain's ability to balance vigilance, flexibility, and agency. Again, in this way, flow restores agency where trauma erodes it, i.e., by reestablishing cognitive control over arousal and uncertainty, the very mechanism that is compromised in PTSD.

Flow's ability to override trauma can be viewed as *behavioral neurofeedback*: a self-generated reorganization of network dynamics. Just as psychedelics and certain neurofeedback protocols temporarily increase cortical entropy and break rigid patterns, flow achieves similar ends through natural, repeatable means. Each episode of deep absorption provides the nervous system with a high-precision demonstration that arousal and safety can coexist, rewriting the brain's threat priors without pharmacological intervention. Repeated experiences expand the system's repertoire of viable states—metastability restored through play, mastery, and meaning.

This framework unites predictive processing, resilience, and the neurobiology of optimal experience. It replaces the notion of trauma as stored injury with a model of trauma as frozen inference. It also clarifies why approaches that emphasize expression, catharsis, or somatic “release” can sometimes help: not because they discharge buried [free] energy, but because they introduce variability and recalibrate precision through novel sensorimotor feedback. Healing, in this light, is not excavation but exploration.

Understanding trauma as a dysregulation of metastability may also dissolve a long-standing paradox in mental health: why so many diverse treatments—exposure therapy, EMDR, mindfulness, exercise, psychedelics, flow-inducing pursuits—can all succeed. Each, in its own way, restores flexible coupling between large-scale networks, quiets maladaptive self-referential loops, and rebalances neuromodulation. The mechanism is not specific content but dynamic reorganization. The nervous system learns to balance oscillation and homogenization, inhibition and excitation.

Framing trauma dynamically does not diminish the suffering it causes, but it grounds that suffering in mechanisms that can be directly addressed. Interventions can target network flexibility, not metaphorical scars. This perspective also guards against pathologizing normal adaptation: most humans recover because their brains retain the capacity for metastable inference. Our task is to support that process, not convince people that their bodies are indelibly marked by pain.

An important limitation of our framework deserves emphasis: the core theoretical claim—that PTSD specifically involves reduced metastability—has not yet been directly tested using established metastability metrics in PTSD populations. The Hellyer et al. (2015) findings on metastability reduction pertain to traumatic brain injury, a neurologically distinct condition, and the conflation of structural connectome damage with the functional dysregulation characteristic of PTSD would be unwarranted. Emerging work on connectome gradient analysis in stress-related disorders offers indirect support, but direct measurement of metastability in PTSD remains a critical gap. Future research should therefore prioritize quantifying metastability as a clinical biomarker in trauma populations specifically tracking signal variability, entropy, and network switching before and after interventions (also see Hancock et al., 2025). Early findings from psychotherapy and flow-based programs suggest that successful recovery coincides with restored variability in resting-state connectivity and increased cross-network integration. These objective indices could, if validated, unify trauma research under a single measurable principle: health equals flexibility.

If the old story held that “the body keeps the score,” the emerging narrative elides somatic chauvinism. It's subtler, and more hopeful. The body does not keep the score; the brain keeps predicting it. When prediction becomes too rigid, experience repeats itself, not because it is stored, but because it cannot yet be reinterpreted. Flow—and other states that expand metastability—offer the nervous system a chance to update its model of the world, to reassign precision where it belongs, and to rediscover safety in uncertainty.

Healing, in the end, is not the erasure of what happened, but the return of movement—within the mind, within the networks, within action, within life itself.
