# Correction: The body does not keep the score: trauma, predictive coding, and the restoration of metastability

**DOI:** 10.3389/fnsys.2026.2015379

**Published:** 2026-10-12

**Authors:** Steven Kotler, Michael Mannino, Glenn Fox, Karl Friston

**Affiliations:** 1 Florida Atlantic University, Boca Raton, FL, United States; 2 Miami Dade College, Miami, FL, United States; 3 University of Southern California, Los Angeles, CA, United States; 4 University College London, London, United Kingdom

**Keywords:** active inference, free energy principle, metastability, post-traumatic stress disorder (PTSD), predictive processing

In the published article, Geraerts et al. (2007) was described as establishing that suggestion can produce damaging false memories and somatic feelings of trauma. The study examined corroboration of reported memories and did not establish the claim as stated. A correction has been made to paragraph 14.

The original text read:

“No one should dispute that traumatic memory is qualitatively different from other forms of memory, and that memories can emerge after years of being non-remembered. Our challenge to van der Kolk, however, stems from: (1) the book's near-complete dismissal of research on false memory syndrome, which shows the manifold ways in which suggestion can indeed lead to damaging false memories and somatic feelings of trauma (Geraerts et al., 2007), and (2) the need to recognize that when traumatic memories emerge in response to cues, it is still cortical and subcortical systems in the brain that are triggering the subsequent states in the body.”

The corrected text reads:

“Memories can emerge after years of being non-remembered, but the experience of remembering and the accuracy of what is remembered are different questions. Geraerts et al. (2007) found that continuously remembered childhood sexual abuse and memories unexpectedly recalled outside therapy were more likely to be corroborated than anticipated memories recovered in therapy. These findings invite caution about the circumstances of recall. They do not demonstrate that suggestion produces somatic feelings of trauma, and lack of corroboration does not establish that a particular memory is false. For the present argument, the distinction matters: the intensity of a bodily response cannot, by itself, settle the historical accuracy of the experience it accompanies.”

In the published article, the review of 54 studies and the 65.7% resilience estimate were incorrectly attributed to Bonanno et al. (2015). The correct source is Galatzer-Levy, I. R., Huang, S. H., and Bonanno, G. A. (2018). Trajectories of resilience and dysfunction following potential trauma: a review and statistical evaluation. *Clin. Psychol. Rev*. 63, 41–55. doi: 10.1016/j.cpr.2018.05.008. This should have been cited as Galatzer-Levy, Huang, and Bonanno (2018). A correction has been made to paragraph 16.

The original text read:

“A systematic review of 54 studies found the resilient trajectory in approximately 65.7% of cases across populations (Bonanno et al., 2015).”

The corrected text reads:

“A review and statistical evaluation of 54 studies found resilience to be the most common trajectory, averaging 65.7% across the populations examined (Galatzer-Levy et al., 2018).”

In the published article, the findings concerning threat-related hyperconnectivity and amygdala/medial prefrontal graph measures were incorrectly attributed to Engdahl et al. (2010). The correct source is Dunkley, B. T., Pang, E. W., Sedge, P. A., Jetly, R., Doesburg, S. M., and Taylor, M. J. (2016). Threatening faces induce fear circuitry hypersynchrony in soldiers with post-traumatic stress disorder. *Heliyon* 2:e00063. doi: 10.1016/j.heliyon.2015.e00063. This should have been cited as Dunkley et al. (2016).

A correction has been made to paragraph 5.

The original text read:

“MEG studies, for instance, found hyperconnectivity during threat processing in posterior cingulate and right parietal regions, with increased node strength and clustering in the amygdala and medial prefrontal cortex, suggesting reorganization rather than blanket rigidity (Engdahl et al., 2010).”

The corrected text reads:

“An MEG study, for instance, found hyperconnectivity during threatening-face processing in posterior cingulate and right parietal regions, with increased node strength and clustering in the right amygdala and medial prefrontal cortex, suggesting reorganization rather than blanket rigidity (Dunkley et al., 2016).”

In the published article, Bomyea and Amir (2011) was incorrectly described as showing that reduced working-memory capacity predicted intrusive-memory severity after accounting for anxiety and depression. The experiment manipulated inhibitory-control demands in undergraduates and assessed intrusive thoughts during a thought-suppression task. A correction has been made to paragraph 22.

The original text read:

“In addition, recent evidence from cognitive neuroscience also challenges the idea that trauma is stored in the body, showing instead that post-traumatic symptoms arise when executive networks lose the capacity for cognitive control. Bomyea and Amir (2011) found, in an analog sample, that reduced working memory capacity, a core marker of cognitive control, predicted the severity of intrusive memories even after accounting for anxiety and depression. Although this finding awaits replication in clinical PTSD populations, it is consistent with the broader principle that executive dysfunction mediates intrusion severity. Trauma's persistence thus reflects a breakdown of top-down regulation rather than a bottom-up somatic residue: when prefrontal systems can no longer suppress or update threat representations, sensory and interoceptive signals dominate belief updating, creating the illusion that the body “keeps score.” PTSD, in this sense, represents a collapse of executive precision and network metastability—the brain's predictive hierarchy loses its ability to maintain balance and correct its own errors through overt or covert action.”

The corrected text reads:

“Cognitive-control research offers another way to understand why unwanted thoughts persist. Bomyea and Amir (2011) randomly assigned undergraduate participants to working-memory training with high or low inhibitory-control demands. Those in the high-demand condition showed greater improvement in working-memory performance and fewer intrusions during a subsequent thought-suppression task. The finding suggests that strengthening inhibitory control can change the persistence of unwanted thoughts in this experimental setting; it does not establish the cause of intrusive memories in PTSD. In the language of our framework, it raises a possibility: stronger control might give the system greater freedom to revise entrenched threat expectations. Whether this process alters metastability in PTSD remains an empirical question.”

In the published article, the initial discussion of Hellyer et al. (2015) did not explicitly identify its traumatic-brain-injury population and extended its findings to psychological trauma too definitively. The revised paragraph distinguishes the empirical finding from the proposed application to PTSD. A correction has been made to paragraph 3.

The original text read:

“This rigidity contrasts with a healthy brain's metastable dynamics, where neuronal networks continually integrate and segregate in response to context. This allows neuronal dynamics to explore multiple (unstable) interpretations of the world. Hellyer and colleagues demonstrated that metastability is a hallmark of cognitive flexibility: the capacity for neural coalitions to assemble transiently and adapt quickly. Using both empirical and computational approaches, Hellyer et al. (2015) showed that reduced metastability arising from damage to the structural connectome was associated with diminished cognitive flexibility and impaired information processing. Trauma erodes this fluidity, trapping the brain in narrow basins of fear and defensive salience. To restore mental health is not about “releasing” stored emotion but reestablishing dynamic equilibrium enabling the brain's ability to move with graceful agility over a landscape of beliefs, commitments and intentions.”

The corrected text reads:

“This rigidity contrasts with metastable dynamics, in which neural networks transiently integrate and segregate as conditions change. Using empirical and computational approaches, Hellyer et al. (2015) found reduced metastability following traumatic brain injury, associated with structural connectome damage, diminished cognitive flexibility, and impaired information processing. These findings concern physical brain injury, not psychological trauma or PTSD. Yet they suggest a question: could persistent threat also narrow the brain's repertoire of available states? We propose this as a theoretical extension. Its relevance to PTSD depends on testing whether altered metastable dynamics accompany persistent symptoms and whether recovery involves a return of context-sensitive flexibility.”

In the published article, Walter et al. (2023) was presented as emerging support for restoration of metastability through flow. The trial concerned major depressive disorder; it did not measure metastability or establish flow as the mediator of improvement. A correction has been made to paragraph 20.

The original text read:

“Clinically, this dynamic restoration is beginning to find empirical support. In a randomized controlled trial, Walter et al. (2023) assigned 96 active duty service members to 6 weeks of either surf therapy or hike therapy. Both activities are known to produce flow (Kotler, 2014). Both groups showed significant improvement in depression over time, and notably, surf therapy participants were more likely to achieve remission from major depressive disorder at three-month follow-up—though the two groups did not differ significantly at post-program. These results are encouraging but warrant caution: the comparable short-term trajectories suggest that generic factors such as exercise, nature exposure, and social connection may contribute substantially, and the specific contribution of flow-state neurobiology cannot yet be isolated from these confounds. Neurobiologically, such experiences likely downregulate the default mode network, reengage executive circuits, and recalibrate threat detection. The brain, forced to navigate rapid feedback in a safe but challenging environment, relearns how to move.”

The corrected text reads:

“Activity-based interventions offer a setting in which to investigate this possibility. Walter et al. (2023) randomized 96 active duty service members with major depressive disorder to 6 weeks of surf or hike therapy. Both groups improved over time, and surf participants were more likely to achieve remission at 3-month follow-up, although the groups did not differ significantly at post-program. These findings are encouraging, but the clinical change does not identify its mechanism. The trial did not measure metastability or test flow as a mediator, and concurrent treatment was permitted. Exercise, nature exposure, and social connection remain possible contributors. Whether moments of deep absorption add something specific to that process is a question for future research; this trial does not precisely establish a flow-based mechanism of recovery in PTSD.”

The citation to Kotler (2014) has also been removed from this paragraph. Because it has no remaining in-text citation, its entry into the **Reference** section has also been removed.

The original version of this article has been updated.

